# What Pinnipeds Have to Say about Human Speech, Music, and the Evolution of Rhythm

**DOI:** 10.3389/fnins.2016.00274

**Published:** 2016-06-20

**Authors:** Andrea Ravignani, W. Tecumseh Fitch, Frederike D. Hanke, Tamara Heinrich, Bettina Hurgitsch, Sonja A. Kotz, Constance Scharff, Angela S. Stoeger, Bart de Boer

**Affiliations:** ^1^Artificial Intelligence Lab, Vrije Universiteit BrusselBrussels, Belgium; ^2^Sensory and Cognitive Ecology, Institute for Biosciences, University of RostockRostock, Germany; ^3^Department of Cognitive Biology, University of ViennaVienna, Austria; ^4^Chemnitz ZooChemnitz, Germany; ^5^Basic and Applied NeuroDynamics Lab, Department of Neuropsychology and Psychopharmacology, Maastricht UniversityMaastricht, Netherlands; ^6^Department of Neuropsychology, Max-Planck Institute for Human Cognitive and Brain SciencesLeipzig, Germany; ^7^Department of Animal Behavior, Institute of Biology, Freie Universität BerlinBerlin, Germany

**Keywords:** evolution of speech, evolution of music, evolution of language, vocal learning, entrainment, timing, synchronization, seal

## Abstract

Research on the evolution of human speech and music benefits from hypotheses and data generated in a number of disciplines. The purpose of this article is to illustrate the high relevance of pinniped research for the study of speech, musical rhythm, and their origins, bridging and complementing current research on primates and birds. We briefly discuss speech, vocal learning, and rhythm from an evolutionary and comparative perspective. We review the current state of the art on pinniped communication and behavior relevant to the evolution of human speech and music, showing interesting parallels to hypotheses on rhythmic behavior in early hominids. We suggest future research directions in terms of species to test and empirical data needed.

## The human sense of rhythm from a comparative perspective

Humans are particularly vocal and musical animals. They flexibly learn new vocalizations and easily perceive and move to rhythm (Bolton, 1894; Fitch, 2009)^1^. Why do humans show these two traits that have only been described in relatively few other animals? Previous research led to conflicting hypotheses on how evolution has shaped human brains and physiology to produce complex vocalizations (Richman, 1993; Fitch, 2000; Galantucci et al., 2006; Fitch and Jarvis, 2013; Manson et al., 2013). Several contrasting hypotheses also exist on how and why human and other animals' brains can perceive complex rhythmic patterns (Merker et al., 2009; Honing et al., 2012; Merchant and Honing, 2013; Patel and Iversen, 2014; Ravignani et al., 2014a). Crucially, these hypotheses differ on assumptions about social structure, ecological conditions, and audio-motor abilities present in early hominids, also providing discordant predictions on rhythm and vocal learning skills in different living species (for reviews see Ravignani et al., 2013a, 2014a; Iversen, 2016; Wilson and Cook, 2016). An influential hypothesis in the field, the vocal learning—beat perception and synchronization hypothesis (Patel, 2006), states that vocal production learning (VPL) is a prerequisite for species to be able to extract a *pulse* from periodic acoustic events (like an internal metronome), and use this inferred pulse to synchronize movements to these external events in a predictive and flexible way (*rhythmical entrainment*). In fact, neural pathways between auditory and motor areas of the brain, which originally evolved for VPL, would also enable precisely timed movements to sounds (Kuypers, 1958, 1973; Jürgens et al., 1982). Only a few species are capable of VPL: that is, to modify existing vocalizations and to imitate novel sounds not belonging to their innate repertoire (Janik and Slater, 2000; Van Parijs et al., 2003). Humans, bats (Boughman, 1998; Knörnschild et al., 2010; Vernes, 2016), elephants (Poole et al., 2005; Stoeger et al., 2012), seals (Ralls et al., 1985), dolphins (Reiss and McCowan, 1993; Favaro et al., 2016), and whales (Foote et al., 2006), together with many bird species (Marler, 1970; Todt, 1975; Marler and Peters, 1977; Scharff and Nottebohm, 1991), have been shown capable of vocal learning (Schusterman, 2008; Petkov and Jarvis, 2012; Nowicki and Searcy, 2014).

Model species can be used to test hypotheses on how our ancestors evolved the neuropsychological prerequisites underpinning speech and music (see also Vernes, 2016). One can either pick model species, which are closely related to humans, and hence should share a specific trait by common ancestry (*homology*), or species that have a similar socio-ecology to humans, and hence independently evolved a similar trait by convergent evolution (*analogy*). If a living animal (i) shares much of its evolutionary history with humans, or (ii) was exposed to environmental conditions and evolutionary pressures similar to early hominids, then commonalities in selected behavioral traits may exist between the two (Fitch, 2010, 2014). This comparative approach is extremely powerful as a way of addressing questions such as (a) how humans acquired complex rhythmic and vocal imitation capacities, (b) why distantly related species but not our closest primate relatives evolved these capacities. Several biological factors may provide an answer to these questions, including brain anatomy, body morphology, social structure, habitat, and ecology. Hence suitable model species to investigate rhythm and VPL in our human lineage should, first and foremost, exhibit rhythm and VPL, and possibly be as close as possible to humans in anatomical, ecological, and evolutionary terms. To test the vocal learning—beat perception and synchronization hypothesis against alternative ones, we suggest below why pinnipeds—including vocal and less vocal species—provide an excellent group of model species.

## Pinnipeds: more vocally flexible than primates, phylogenetically closer to humans than birds

Traditionally, VPL and rhythmic behavior have been investigated in primates, parrots or songbirds. Monkeys and non-human apes, like chimpanzees, are evolutionarily and cognitively close to humans, but exhibit limited vocal imitation and rhythmic patterning skills (Janik and Slater, 1997; Ravignani et al., 2013a; Repp and Su, 2013; see Gamba et al., 2016, for timing in lemur singing). In contrast, many bird species are excellent at learning to imitatively produce new vocalizations (Petkov and Jarvis, 2012). Moreover, when tested on non-vocal rhythmic tasks requiring precise temporal coordination, birds outperform primates, although direct primate-avian comparisons on identical tasks are lacking at present (Nagasaka et al., 2013; Hoeschele et al., 2015; Benichov et al., 2016; ten Cate et al., 2016). However, the last common ancestor of birds and humans lived about 300 million years ago (Kumar and Hedges, 1998), and birds have evolved a vocal production system (the syrinx) quite different from the human larynx (Fitch, 2010; Elemans et al., 2015). Hence, primates and birds each have only one of the desirable features to understand rhythm and VPL: non-human primates are evolutionary close to humans but exhibit scarce rhythm and VPL capacities, while birds have rhythmic capacities and VPL but are evolutionary distant from humans.

A third taxonomic group, previously overlooked in comparative research on human evolution (cf. Cook et al., 2013; Rouse et al., 2016), may be the solution to this conundrum. Pinnipeds exhibit VPL and rhythmic abilities (Table 1), and as mammals they are evolutionary closer to humans than birds: the last common ancestor of humans and pinnipeds lived about 65 MY ago (O'Leary et al., 2013). This clade includes more than 30 species of semiaquatic mammals divided in three families: Phocidae (e.g., harbor and gray seals), Otariidae (e.g., California sea lions and Cape fur seals), and Odobenidae (walruses). Pinniped phylogeny is controversial. However, recent molecular evidence suggests that the first split, separating Phocidae from other pinnipeds, occurred 33 MY ago (Arnason et al., 2006). This relatively old common origin—compare it with the 33 MY between humans and e.g., capuchin monkeys (Glazko and Nei, 2003), has provided ample time to adapt to many different ecological niches and environmental constraints. Accordingly, pinniped species exhibit variation in VPL capacities, social organization, mating systems, and habitats (Table 1). These dimensions conveniently have anthropological equivalents, each of them deemed crucial for at least one hypothesis on the evolution of speech and music (Fitch, 2000; Hagen and Bryant, 2003; Patel, 2006; Hagen and Hammerstein, 2009; Merker et al., 2009; Petkov and Jarvis, 2012; Merchant and Honing, 2013; Patel and Iversen, 2014; Ravignani, 2014; Ravignani et al., 2014a,b; for a comparative definition of speech).

**Table 1:** **Features of human speech and music (first column) are related to findings in pinniped biology (second column) to draw comparative conclusions and suggest further research (third column)**.

**Human speech or music feature**	**Corresponding trait in pinnipeds' biology/behavior/cognition**	**Implications, questions, and hypotheses**
**A**. Humans can imitate vocalizations and **speak**.	Harbor seals can imitate human speech (Ralls et al., 1985).	Harbor seals can modify their vocal behavior through experience.
**B**. Speech sounds are produced by the **larynx** and filtered by the upper vocal tract.	Pinnipeds have a similar vocal anatomy to humans, producing some vocalizations with their larynx at similar angle with respect to airflow (Schneider et al., 1964).	Same larynx-passive framework (see Fitch, 2000) applicable to humans and pinnipeds (comparing vocally flexible to inflexible pinniped species).
**C**. **Upper vocal tract** is used to produce vowels and consonants via formant filtering.	Human-raised harbor seal's vocalizations match spectral features of human speech (Ralls et al., 1985), possibly produced using the upper vocal tract (Spasikova et al., 2008).	Similar neural mechanism enabling vocal tract reconfiguration and formant filtering in humans and seals.
**D**. Maternal interactions affect the development of **infant speech**.	Mothers recognize and adapt to their pup's vocalizations, which vary over time (Sauvé et al., 2015a,b).	Development of vocalizations may be flexible and interactive rather than strongly innate.
**E**. Languages are variable (e.g., **dialects**).	Geographic variation in vocal repertoires due to genetics and learning (Van Parijs et al., 2003; Reichmuth and Casey, 2014).	Some vocalizations are socially learnt and modified.
**F**. Human **brains** can readily process speech and music.	Mammal brains have similarities due to relatively recent common ancestry.	Common, evolutionary old brain areas (e.g., subcortical structures) are expected to enable rhythm perception and production (Kotz and Schwartze, 2010; Knolle et al., 2012).
**G**. Humans accurately **entrain** across tempos and modalities.	Highly developed rhythmic skills in a supposedly vocally inflexible California sea lion (Cook et al., 2013; Rouse et al., 2016).	Current evidence from sea lions not consistent with the “vocal learning—beat perception and synchronization” hypothesis (cf. Patel, 2014). Other biological factors, such as social organization or mating system, may affect rhythmic skills.
**H**. Human cognitive capacities for speech and music are tested in **operant tasks**.	Pinnipeds are reasonably easy to train using operant conditioning techniques (e.g., Schusterman, 2008; Stansbury et al., 2015).	Direct comparison of human and pinniped abilities in music and cognitive experiments is possible.
**I**. Accurate **timing** is crucial in speech and music production.	(1) Multilevel temporal information is important in the production of natural vocalizations (Schusterman, 1977; Riedman, 1990; Van Parijs et al., 1999, 2003; Spasikova et al., 2008); (2) pinnipeds show good acoustic temporal resolution (Mulsow and Reichmuth, 2007) and visual interval timing abilities (Heinrich, 2013).	Vocally-flexible (harbor seals) or social (California sea lions) pinniped species should exhibit enhanced timing skills, providing support for one of the many evolutionary hypothesis.
**L**. Humans have excellent **rhythm perception**.	(1) Mammalian hearing; (2) Pinniped whiskers can precisely sense periodic mechanical stimuli (Mills and Renouf, 1986).	Pinnipeds may have multimodal (i.e., whiskers' kinaesthetic) sound/rhythm perception capacities (sounds are periodic mechanical stimuli, which may be sensed through whiskers).
**M**. **Meter:** Auditory experience modulates innate biases for grouping multiple sounds.	Some species experience an extremely variable acoustic environment generated by conspecifics' vocalizations (Riedman, 1990). This auditory experience could affect the developmental trajectories of grouping and top-down perception of sounds (Toro, 2016).	Vocal learners (e.g., harbor seals) should perceive meter more readily than vocally-inflexible species (e.g., California sea lions).
**N**. **Percussions** may be the first human musical instruments.	Harbor seals perform water slapping displays, drumming a series of hits on the water surface (Riedman, 1990).	Possible functional analogy between harbor seals' slapping and early humans' drumming.
**O. Sexual dimorphism:** Hypotheses on the origins of speech, language, and music vary in the amount of between-sex differences hypothesized in early hominids.	Pinniped species exhibit a broad range of mating systems (ranging from polygyny to serial monogamy) and forms of sexual dimorphism (male and female are almost indistinguishable in some species and drastically different in other species) (Riedman, 1990).	Dependent on the particular evolutionary hypothesis (Iversen, 2016), pinniped species should exhibit positive, negative, or no correlation between sexual dimorphism in vocal repertoire and rhythmic skills.
**P. Working memory:** Auditory short-term (working) memory is crucial in human speech and music perception.	Some pinnipeds, such as California sea lions, have particularly good visual and auditory working memory (Schusterman and Kastak, 2002). Pinnipeds' auditory working memory might in some cases even exceed that of non-human primates (Fritz et al., 2005, Scott et al., 2012).	Additional comparative research should confirm highly developed auditory working memory in some pinniped species, making them promising model species for speech and musical rhythm.

Notably, among the pinnipeds, harbor seals (*Phoca vitulina*) exhibit an excellent trade-off between VPL abilities and phylogenetic proximity to humans: among vocal learners, harbor seals have the closest vocal apparatus to humans (Schneider, 1962; Schneider et al., 1964; Ralls et al., 1985; Fitch, 2000; Table 1A,B). A human-raised harbor seal has even learned to imitate some human words and phrases (Ralls et al., 1985; Table 1C). So far, harbor seals have not been tested for rhythmic entrainment abilities; however, another pinniped species, the California sea lion (*Zalophus californianus*) was shown capable of non-vocal audio motor synchronization with precision previously exhibited only by avian species and humans (Cook et al., 2013; Rouse et al., 2016; Table 1G). With these few exceptions, pinniped communication, rhythm, and human speech have mostly remained unconnected areas of research until now. However, a lot of information is available on pinnipeds' natural vocal behavior, making the comparative study of pinniped communication and human speech a field ripe for research. We suggest that pinnipeds are ideal species to understand human speech, rhythm, and complex VPL at different levels (including physiology, behavior, neurobiology, and genetics). Pinnipeds' vocal anatomy, brain evolutionary history, socio-ecology, and broad range of environmental conditions conveniently map to human biology (Schneider, 1962; Ralls et al., 1985; Riedman, 1990; Van Parijs et al., 1999, 2003; Schusterman, 2008; Cook et al., 2013; Sauvé et al., 2015a,b; Table 1).

Then, why do humans and harbor seals produce flexible vocalizations? Taking ultimate and proximate causes into account and adopting a comparative approach (Table 2), we suggest several strands of empirical research in pinnipeds, which can shed light on the evolution of human rhythmicity.

**Table 2:** **The question of why a particular behavioral trait, such as vocal production learning, exists in a species can be answered taking ultimate and proximate causes into account (Tinbergen, 1963)**.

	**Humans**	**Pinnipeds**
Mechanism	In humans the mechanisms underlying speech production are increasingly well understood by studying brain areas e.g., auditory and motor cortices, basal ganglia (Kung et al., 2013), vocal folds' dynamics, and sound articulation in the upper vocal tract (Fitch, 2000).	Likewise, an increasingly compelling hypothesis is that harbor seals may produce flexible vocalizations via human-like laryngeal vibrations and finely controlled vocal tract filtering (Schneider, 1962; Schneider et al., 1964; Spasikova et al., 2008; Table 1A–C).
Ontogeny	The ontogeny of human speech production is studied by tracking how the linguistic input infants receive from birth influences and shapes the uttering of first words.	The ontogeny of vocal production in harbor seals is quite complex: early developmental influences due to mother-infant communication (Sauvé et al., 2015b) seem to complement later social interactions (Riedman, 1990; Table 1D,E).
Function	Contrasting hypotheses on the original function of human speech abound, ranging from a primate-like lip-smacking social display, later exapted for communication, to mate attraction via production of complex vocalization, as in songbirds (see Fitch, 2010).	Vocal behavior in harbor seals is involved in male-male competition (Hanggi and Schusterman, 1994), mother-infant interaction (Sauvé et al., 2015a,b), individual recognition, sexual and territorial advertisement, or lek (i.e., group competitive) displays (Hayes et al., 2004); VPL may have evolved under functional pressure for one of these functions (Table 1E,I,M).
Phylogeny	Current evidence suggests that humans were the only ones who acquired speech (Fitch, 2000, 2010) among the ancestors of living apes, instead of the alternative possibility that all apes but humans have lost an ancestral proto-speech.	Phylogeny of VPL in seals is more uncertain: phocids and walruses (*Odobenus rosmarus*) are vocal learners but sea lions seem not to be (Schusterman, 2008; Schusterman and Reichmuth, 2008; Reichmuth and Casey, 2014; Stansbury et al., 2015).

## Future research: what species to test next, and in which tasks?

### Vocal production learning

Pinnipeds produce many types of vocalizations, which can be recorded in air, enabling acoustic data collection with precise individual identification. Research in harbor seals, building on existing evidence on vocal imitation (Ralls et al., 1985), should investigate their ability to learn vocalizations (i) over developmental phases, and (ii) from each other in a social network (Janik and Slater, 2000; Tyack, 2008; Table 1A–F). This will reveal how seal vocalizations are imitated and transformed (Fitch, 2015b) similarly to human speech. In parallel, vocal flexibility in Otariids should be investigated across species, testing their ability to imitate new sounds. This will hopefully provide clear support for or against VPL capacities in this pinniped family considered, until now, the least vocally flexible. While performing this research, it will be important to keep an open-minded attitude toward vocal learning, as this seems to be a graded ability rather than an all-or-none trait (Petkov and Jarvis, 2012; Fitch, 2015a).

Comparative vocal and brain anatomy in pinnipeds can be fruitful strands of research (Table 1B,C,F). The angle of vocal folds with respect to the tracheal air stream is 76° (degrees) in harbor seals, while 17.5° in sea lions (Schneider et al., 1964). This suggests sea lions have a vocal folds' angle closer to elephants (45°); harbor seals' angle instead is closer to humans (90°) than to sea lions (Herbst et al., 2013). Does this difference in vocal anatomy map to a difference in types of sounds produced or just modalities of sound production?

Neuroanatomy may constitute a fruitful research avenue to understand the mechanisms behind successful entrainment in California sea lions. Although the shape of their brain is similar to that of other carnivores, analyses of brain folding show remarkable differences. In particular, California sea lions have more secondary folds and sulci, and a radically different pattern of folds and fissures than other carnivores such as canids, e.g., dogs, wolves, coyotes, and mustelids e.g., minks (Montie et al., 2009). This suggests evolutionary pressures and potentially similar mechanisms increased the size of the neocortex in sea lions showing an interesting parallel to human evolution. A further open question is how the evolution of different brain structures relates to VPL (Patel, 2014) and social organization across pinniped species. Comparative brain anatomy and imaging will elucidate whether evolutionary old brain circuits subserving VPL are still present in vocally inflexible pinnipeds, such as sea lions (Patel, 2014).

### Interval timing and synchronization

Timing experiments often investigate the attentional and cognitive processes involved in perceiving or estimating single time intervals, either independently or by comparison with a second interval (Grondin, 2010). These experiments have, for instance, shown similarities and differences between humans and other primates in estimating *single interval* durations in the visual and auditory modality (Merchant et al., 2003; Zarco et al., 2009; Mendez et al., 2011). In pinnipeds, recent data show that a harbor seal and a Cape fur seal (*Arctocephalus pusillus*) can accurately discriminate time intervals in the visual modality (Heinrich, 2013; Table 1I). In contrast, rhythm refers to the structure of *multiple durational events*, i.e., sequences of time intervals. Hence, single-interval timing research is essential (Merchant and Honing, 2013) though not enough to understand rhythm perception: in fact, perception of one interval influences perception of adjacent intervals (McAuley, 2010). Studying perception, reproduction, and entrainment to isochronous (metronome-like) sequences is the first step when moving from timing to rhythm research. In entrainment experiments, humans and other animals are tested on their ability to synchronize their movements to an external visual or auditory metronomic stimulus. Synchronization can arise spontaneously or be trained by the experimenter. Crucial experimental criteria for successful synchronization are: (i) flexibility, i.e., comparable performance at different tempos, (ii) multimodality i.e., ability to synchronize one's behavior in a sensory modality different from that of the external stimulus, and (iii) predictive rather than reactive behavior, i.e., zero or negative asynchrony, and unperturbed performance when one beat is missing (Patel et al., 2009a,b).

Extending previous entrainment studies in otariids (Cook et al., 2013; Rouse et al., 2016), harbor seals' and walruses' ability to entrain should be tested (Tables 1G,L). Successful synchronization in one of these vocal learners (Reichmuth and Casey, 2014) would provide an important data point in support of the VPL—rhythm link (Patel, 2006). Useful out-groups for synchronization experiments could be non-pinniped Canoidea, like dogs, exhibiting almost no VPL (Janik and Slater, 1997; Taylor et al., 2009). Harbor seals' and walruses' inability to synchronize would not refute Patel's hypothesis. However, failure to synchronize would refute alternative hypotheses, postulating individual territorial advertisement or lek displays as crucial factors for the evolution of rhythm (Hagen and Hammerstein, 2009; Ravignani, 2014).

### Natural isochronous behavior and perception of isochrony

As flexible synchronization requires the ability to represent an isochronous pulse (Iversen and Balasubramaniam, 2016), pinnipeds should be tested on their ability to discriminate between isochronous and non-isochronous temporal patterns. In birds, the ability to recognize isochronicity in acoustic sequences seems to positively correlate with VPL: pigeons perform much worse (Hagmann and Cook, 2010) than other birds capable of VPL, like zebra finches and starlings (Hulse et al., 1984; van der Aa et al., 2015). If this can be generalized, one would analogously expect harbor seals and walruses tested in comparable setups to outperform e.g., California sea lions and Cape fur seals. Finally, pinniped species naturally showing isochronous vocal behavior may be particularly promising to test in order to ascertain how VPL and natural isochronous behavior affect the ability to entrain. While vocalizations in the vocally inflexible Australian and California sea lions can be quite regular, the vocally flexible harbor seals vocalize with much less temporal regularity (Schusterman, 1977; Charrier et al., 2011).

### Meter perception, grouping, and auditory experience

Meter provides an additional dimension to rhythmic patterns, where individual events in time have different perceptual or acoustic “weights.” *Meter* is defined as hierarchical organization of temporal events (McAuley, 2010). Meter corresponds to hearing events in time as related, forming structured patterns, e.g., the alternation of weak/strong beats in music and stressed/unstressed syllables in speech (Fabb and Halle, 2012). Meter perception can occur in sequences of stimuli that are acoustically identical (Brochard et al., 2003), or instead based on stimuli that alternate in duration, frequency, or amplitude (McAuley, 2010; Toro and Nespor, 2015; Geambasu et al., 2016; Hoeschele and Fitch, 2016).

Humans can perceive a range of metrical patterns but are biased toward specific metrical grouping patterns, partially depending on their native language (Iversen et al., 2008). In particular, a few perceptual laws, such the iambic-trochaic law (de la Mora et al., 2013), may explain most of rhythmic grouping in speech and music (Figure 1 in Supplementary Material). Rats, for instance, exhibit experience-modulated grouping biases: Like humans, they spontaneously group sequences when sounds alternate in pitch, but do not when sounds alternate in duration (de la Mora et al., 2013). However, rats can *learn* to group sounds of alternating durations: if exposed to short-long sequences, they will show the corresponding iambic bias when tested; if familiarized with long-short, rats will prefer trochaic grouping (Toro and Nespor, 2015).

Meter perception should be investigated across pinnipeds (Table 1M). As grouping is influenced by auditory experience, we would expect pinnipeds with a varied conspecific auditory input, like harbor seals, to require little training to discriminate metrical patterns. After probing pinnipeds' predictive timing by having them produce behavioral responses, temporal expectations could be explored by directly tapping into perception. Adapting non-invasive electrophysiology originally developed for humans and non-human primates, one could record event-related potentials corresponding to click sounds repeating at a constant rate, and compare these potentials to those evoked by click trains containing missing clicks or metrically-structured (accented) clicks (Rothermich et al., 2010; Schmidt-Kassow et al., 2011; Schwartze et al., 2011; Honing et al., 2012; Selezneva et al., 2013; Celma-Miralles et al., 2016; Cirelli et al., 2016).

### Percussive behavior in harbor seals

Empirical evidence from human archeology, ethnomusicology and African apes' behavior suggest that percussion may have been the first form of musical expression in our hominid ancestors (Arcadi et al., 1998; Morley, 2003; Fitch, 2009). What was the function of rhythmic drumming in early hominids? A behavioral display in harbor seals may help answer this question: Accompanying vocalizations, harbor seals “drum” on the water, repeatedly slapping their flippers on the sea surface (Riedman, 1990; Wahlberg et al., 2002). Once again, hypotheses on the function of this slapping behavior mimic hypotheses proposed for human drumming (e.g., Kirschner and Tomasello, 2009). Slapping in harbor seals may function as signal in agonistic sexual displays (Riedman, 1990), or as a form of intrasexual competition to attract females (Nikolich, 2015). Another hypothesis regards water drumming as a form of territorial advertisement in agonistic contexts: in fact, during the breeding season, male seals produce slaps in response to other males either intruding a territory, or challenging an intruder (Hayes et al., 2004). Water slapping may hence indirectly play a role in establishing and maintaining dominance hierarchies, similar to chimpanzees' drumming (Arcadi et al., 1998; Ravignani et al., 2013b).

One hypothesis we suggest is that vocal displays and drumming displays may have the same territorial function but be used complementarily. Seals' slaps cover a different frequency band than, and have dramatically different durations from, roars. Slaps last about 0.002 s, contain most frequency between 5 and 20 kHz, and have (in-water) source intensity of 166–199 dB (Wahlberg et al., 2002). In contrast, roars last 2–3 s, are centered at frequencies of 200–300 Hz and have 150 dB intensity (Hayes et al., 2004). How far can each of these sounds travel so that they are still audible by seals? At 200 Hz, seals' hearing threshold is 32 dB (82 dB underwater); the sensitivity is much higher between 5 and 20 kHz, reaching 1–29 dB (60–62 dB in water; Reichmuth et al., 2013). Hence (1) slaps carry much farther than roars, (2) even if a slap and a roar reach a seal with the same sound intensity, a slap will be more conspicuous: slap might be perceived up to 30 times louder than a roar, and (3) slaps could be in principle perceived visually (Nikolich, 2015). Seals' water slaps hence seem to mimic many features of early human's territorial advertisement, which have been hypothesized to underlie the evolution of human musicality (Hagen and Hammerstein, 2009).

Future research should record individuals over time to: (i) analyse the fine-grained temporal structure of series of slaps (Babiszewska et al., 2015); (ii) test whether drumming and its temporal parameters are socially learnt, and if so (iii) compare the social dynamics of two transmitted rhythmic behaviors, across modalities (vocalizations vs. slapping), and (iv) relate water-slapping to similar percussive behaviors present in humans and chimpanzees (Fuhrmann et al., 2014; Whiten, 2015; Table 1N). Collection of slapping data will enable to test hypotheses postulating group and mating displays as necessary evolutionary steps toward human musicality (Fitch, 2009; Merker et al., 2009). In fact, if harbor seals' slaps show strong temporal interdependence between individuals, successful entrainment experiments in this species would support the hypothesis that rhythm may have evolved in humans as by-product of temporally-intertwined group displays (Merker et al., 2009).

## Conclusions

Researchers of human evolution and pinniped communication have been suggesting, unbeknownst to each other, similar hypotheses for the evolution of human speech and music, on the one hand, and pinnipeds' vocal displays and non-vocal communication, on the other hand. Advocating the comparative method and the distinction between proximate and ultimate questions, we have shown how animal research can help formulate and test hypotheses about the evolution of human speech and music. We have briefly reviewed previous findings in pinniped biology, explicitly pointing out their relevance to the human sense of rhythm in music and speech. We have discussed crucial questions that pinniped research should address empirically, possibly using comparable stimuli, tasks, and analysis techniques across species, ultimately shedding light on the origins of rhythmic behaviors in humans.

## Author contributions

Andrea Ravignani wrote the manuscript. All authors provided ideas and edited the manuscript.

## Funding

Andrea Ravignani was supported by FWO grant V439315N (to Andrea Ravignani), and European Research Council grant 283435 ABACUS (to Bart de Boer).

### Conflict of interest statement

The authors declare that the research was conducted in the absence of any commercial or financial relationships that could be construed as a potential conflict of interest.

## Footnotes

^1^Rhythm is defined as a “serial pattern of durations marked by a series of events” (McAuley, 2010. pp. 166).
