# Supplementary material for: What Pinnipeds Have to Say about Human Speech, Music, and the Evolution of Rhythm
Source: Front Neurosci. 2016 Jun 20;10:274. doi: 10.3389/fnins.2016.00274 (PMC4913109; doi:10.3389/fnins.2016.00274)
Supplement: Supplementary file 1 [file Presentation1.PDF]

What pinnipeds have to say about human speech, music, and the evolution of rhythm

Andrea Ravignani[1,2], W. Tecumseh Fitch[3,#], Frederike D. Hanke[2,#], Tamara Heinrich[2,#], Bettina Hurgitsch[4,#], Sonja A. Kotz[5,6,#], Constance Scharff[7,#], Angela S. Stoeger[3,8,#], Bart de Boer[1]

Supplementary Figure

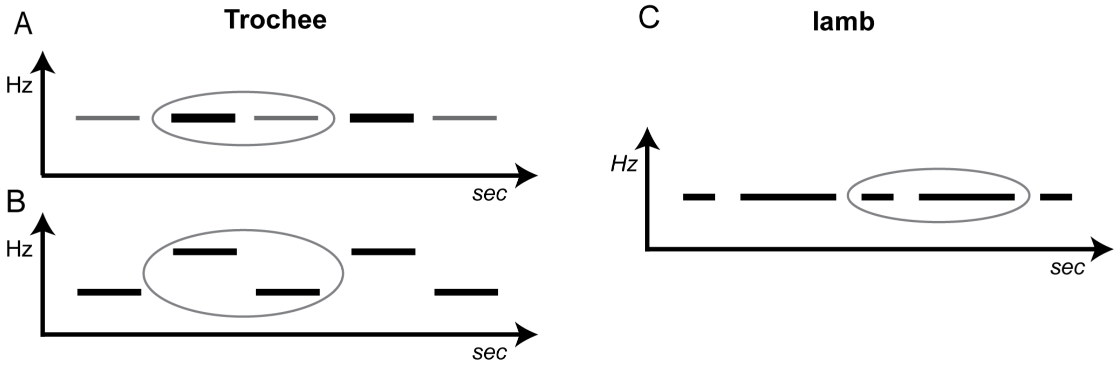

Figure 1. If presented with sequences of equal-length sounds with (A) alternating intensity (weak-strong-weak) or (B) pitch (low-high-low), humans seem to have an innate tendency to group them as *strong-weak* (grouping represented by grey oval). This is called *trochaic* grouping. If presented with sequences of (C) two alternating durations (short-long-short), some humans, depending on their native language (Iversen et al., 2008), group sounds as *weak-strong* (corresponding to short-long). This is called *iambic* grouping.
